# Multidisciplinary care for patients with HCC: a systematic review and meta-analysis

**DOI:** 10.1097/HC9.0000000000000143

**Published:** 2023-04-26

**Authors:** Karim Seif El Dahan, Annika Reczek, Darine Daher, Nicole E. Rich, Ju Dong Yang, David Hsiehchen, Hao Zhu, Madhukar S. Patel, Maria del Pilar Bayona Molano, Nina Sanford, Purva Gopal, Neehar D. Parikh, Adam C. Yopp, Amit G. Singal

**Affiliations:** 1 Department of Internal Medicine, UT Southwestern Medical Center, Dallas, Texas, USA; 2 Department of Internal Medicine, Cedars Sinai Medical Center, Los Angeles, California,USA; 3 Department of Surgery, UT Southwestern Medical Center, Dallas, Texas, USA; 4 Department of Radiology, UT Southwestern Medical Center, Dallas, Texas, USA; 5 Department of Radiation Oncology, UT Southwestern Medical Center, Dallas, Texas, USA; 6 Department of Pathology, UT Southwestern Medical Center, Dallas, Texas, USA; 7 Department of Internal Medicine, University of Michigan, Ann Arbor, Michigan, USA

## Abstract

Given the complexity of managing HCC, professional society guidelines advocate multidisciplinary care (MDC) for patients with HCC. However, implementation of MDC programs requires a significant investment of time and resources. We conducted a systematic review and meta-analysis to enumerate potential benefits of MDC for patients with HCC.

**Methods:**

We conducted a search of the PubMed/MEDLINE and EMBASE databases and national conference abstracts to identify studies published after January 2005 that reported early-stage presentation, treatment receipt, or overall survival among patients with HCC, stratified by MDC status. We calculated pooled risk ratios and HRs for clinical outcomes according to MDC receipt using the DerSimonian and Laird method for random effects models.

**Results:**

We identified 12 studies (n = 15,365 patients with HCC) with outcomes stratified by MDC status. MDC was associated with improved overall survival (HR = 0.63, 95% CI: 0.45–0.88); however, its association with curative treatment receipt was not statistically significant (risk ratio = 1.60, 95% CI: 0.89–2.89) and pooled estimates were limited by high heterogeneity (*I* ^2^ > 90% for both). Studies (n = 3) were discordant regarding an association between MDC and time-to-treatment initiation. MDC was associated with early-stage HCC (risk ratio = 1.60, 95% CI: 1.12–2.29), suggesting possible referral bias contributing to improved outcomes. Limitations of studies also included risk of residual confounding, loss to follow-up, and data preceding the availability of immune checkpoint inhibitors.

**Conclusion:**

MDC for patients with HCC is associated with improved overall survival, underscoring the likely benefit of managing patients with HCC in a multidisciplinary care setting.

## Graphical abstract

**Figure:**
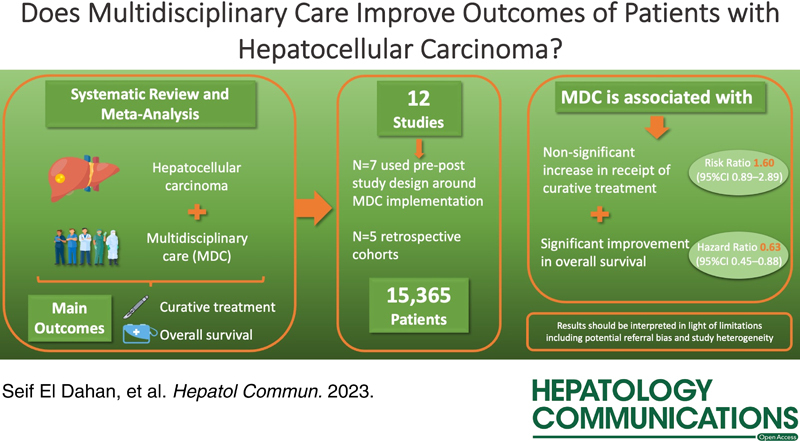

## INTRODUCTION

HCC is the fifth most prevalent cancer globally and claims close to 800,000 lives per year.^1^ It is one of the few cancers with a rising mortality rate in the US and is projected to become the third leading cause of cancer-related mortality by 2030.^2^ The 5-year survival of HCC approaches 70% for early-stage HCC but remains below 20% overall despite recent advances in surveillance and therapeutic options.^3^ This poor survival relates to failures across the cancer care continuum, including ineffective early detection strategies, diagnostic and therapeutic delays, and underuse of guideline-concordant treatment.^4^,^5^

HCC is a complex disease given the near universal presence of underlying advanced chronic liver disease as well as multiple available treatment modalities offered by various treating specialties.^6^ In contrast to prior treatment allocation algorithms in which one treatment was recommended for a specific tumor stage, there are now multiple treatment options to consider based on several factors including tumor burden, liver dysfunction, performance status, transplant eligibility, local expertise, and patient preferences.^6^ Therefore, many expert panels, including the American Association for the Study of Liver Diseases (AASLD) guidance document, advocate for multidisciplinary care (MDC) of patients with HCC.^7^,^8^

There are several models of MDC, ranging from a multidisciplinary tumor board to a fluid referral system between specialists to co-located clinics with multiple specialists being present concurrently.^9^ A multidisciplinary team generally comprises specialists in multiple fields including hepatology, surgical oncology, transplant surgery, interventional radiology, medical oncology, and radiation oncology. Furthermore, large MDC programs often engage other specialties, including palliative care, nutrition, social work, and nursing, as well as the patients in their own care. MDC facilitates communication between specialists to provide more individualized care with the aim of optimizing the diagnosis, treatment, and outcomes of patients with HCC. Indeed, data increasingly indicate that MDC has a beneficial association with the management and outcomes of other cancers.^10^–^12^ MDC can also decrease the number of necessary clinic visits, addressing patient-reported barriers to care including missed work and transportation.^13^ However, implementation of MDC programs often requires a significant investment of time and resources. Tumor boards are typically noncompensated activities, hospitals may not have access to all necessary specialists, MDC clinics can result in fewer patient encounters than would be seen otherwise, and efficient evaluation by multiple providers requires effective patient navigation services.^14^ Therefore, tangible evidence enumerating improvements in clinical outcomes for patients with HCC can be informative for health systems that are contemplating making this investment. To address this need, we conducted a systematic review and meta-analysis of available studies evaluating the potential benefits of MDC for patients with HCC.

## METHODS

This study was conducted following Preferred Reporting Items for Systematic Review and Meta-Analysis (PRISMA) guidelines.^15^

### Search strategy

Two investigators independently performed an electronic-based search of the PubMed, Ovid MEDLINE, and EMBASE databases to identify relevant articles evaluating potential benefits of MDC published between January 2005 and January 2022. The search terms included “liver ca$ OR hepatocellular ca$ OR hepatoma OR HCC” AND “multidisciplinary OR multispecialty OR multidisciplin$ OR multispecial$ OR team.” Manual searches of reference lists were also conducted to identify citations that may have been missed by the electronic-based search. A search of the AASLD, European for the Study of the Liver (EASL), Digestive Disease Week (DDW), and American College of Gastroenterology (ACG) conference abstracts for 2019–2022 was also conducted.

### Study selection and inclusion/exclusion criteria

After removal of duplicate citations, screening of titles, abstracts, and full texts of the remaining citations were conducted by one investigator to generate a list of potentially relevant articles. A second investigator reviewed full texts of articles in this list and included them based on specific criteria. Disagreements and discrepancies were resolved by a third investigator. We included studies that: (1) involved patients with HCC from any etiology; (2) evaluated the clinical impact of a clearly defined MDC; and (3) compared curative treatment receipt or overall survival (OS) between patients with HCC who were under MDC and those who were not. We excluded studies that only reported outcomes among patients who received MDC without a comparator arm. Additional exclusion criteria were as follows: (1) reviews, opinion letters, case reports, or incomplete reports; (2) nonhuman data; (3) lack of original data; and (4) non-English language articles. For studies reporting data on overlapping cohorts, the publication with more complete data was included for analysis.

### Data extraction and quality assessment

Articles meeting inclusion and exclusion criteria were independently reviewed by 2 investigators to extract required information using standardized forms, including cohort size and characteristics, study design, HCC staging system used [ie, Barcelona Clinic Liver Cancer (BCLC) stage, Milan criteria, and TNM staging], type of MDC model (eg, tumor board and co-located clinic), and clinical outcomes of patients with HCC. Clinical outcomes included proportion of patients with early HCC stage detection, time-to-treatment initiation, curative treatment receipt, noncurative treatment receipt, length of follow-up, and OS. Curative treatment was defined as liver transplantation, liver resection, or local ablation, whereas noncurative modalities included transarterial chemoembolization, radiation therapy, and systemic therapy. Study quality and risk of bias were assessed by 2 investigators using a modified National Institutes of Health Study Quality Assessment Tool. Discrepancies were resolved through discussion with a third investigator.

### Statistical analysis

For each study, we calculated a risk ratio (RR) with the exposure being MDC and clinical outcomes being the proportion of patients with early-stage HCC and/or the proportion who underwent curative treatment. For survival, we abstracted HRs when available; if not reported, we recorded median survival for both MDC and control groups. If a HR was reported without a 95% CI, we computed it using the effect estimate and the *p*-value.^16^ We used the DerSimonian and Laird method for random effects models to calculate pooled RR estimates for early-stage HCC and curative treatment receipt and pooled HR estimates for OS.^17^ We used the χ^2^ test of heterogeneity and the inconsistency index (*I* ^2^) to quantitatively determine the extent of heterogeneity between studies. If significant heterogeneity (*I* ^2^ > 50%) was found between studies, we used sensitivity analysis to exclude outlier studies one at a time to assess if this impacted pooled effect estimates. We performed subgroup analyses to explore potential causes of heterogeneity among results. Potential publication bias was evaluated graphically using Begg’s funnel plot and statistically using Egger’s regression test and fail-safe N.^18^–^20^ All data analysis was performed using R software 4.2.1.

## RESULTS

### Study characteristics

Of 2484 potentially relevant titles, 189 were selected for abstract review and 53 for full-text review (Supplemental Figure 1, http://links.lww.com/HC9/A271). After full-text review, 7 articles met inclusion criteria. Searches of annual meeting abstracts yielded 5 additional relevant abstracts, resulting in the inclusion of a total of 12 studies. Egger’s test did not suggest the presence of publication bias for studies assessing early-stage HCC detection (*p* = 0.09), curative treatment receipt (*p* = 0.37), or OS (*p* = 0.29). Similarly, examination of the funnel plot did not suggest publication bias for studies examining the association between MDC and OS. The fail-safe N (ie, number of studies with a null effect that would render the pooled effect size statistically insignificant) was 210 for early-stage HCC detection and 388 for OS.

Characteristics of studies evaluating MDC for patients with HCC are detailed in Table 1. Seven were single-center quasi-experimental studies using a pre-post study design around the time of MDC implementation. The remainder were either single-center (n = 3) or multicenter (n = 2) retrospective analyses comparing patients who received or did not receive MDC during the study period. Most studies were conducted in the US, although there were studies from Turkey (n = 1), South Korea (n = 1), and Australia (n = 1). MDC models included a multidisciplinary conference for imaging review and discussion of management decisions across all studies; however, MDC was additionally defined by evaluation in a co-located multidisciplinary clinic in 2 studies (Yopp and colleagues and Vora and colleagues) and being seen by at least 3 disciplines in the peridiagnostic, pretreatment period in one study (Chirikov and colleagues).

**TABLE 1:** Characteristics of included studies

References	Study location	Study period	Study design	Definition of multidisciplinary care	Number of patients
Chang et al^21^	San Francisco, USA	2000–2006	Pre vs. post, single center	Multidisciplinary management conference	183
Yopp et al^22^	Dallas, USA	2006–2011	Pre vs. post, single center	Multidisciplinary liver tumor clinic+management conference	355
Kani et al^23^ ^a^	Istanbul, Turkey	2007–2013	Pre vs. post, single center	Multidisciplinary management conference	162
Chirikov et al^24^	SEER-Medicare	2000–2007	Retrospective, multicenter	Multispecialty care (3+ disciplines) between 4 wk prediagnosis to week of treatment	2245
Davison^25^ ^a^	Sydney, Australia	2003–2013	Pre vs. post, single center	Multidisciplinary management conference	177
Vora^26^ ^a^	Atlanta, USA	2012–2015	Retrospective, single center	Multidisciplinary liver tumor clinic	447
Agarwal et al^27^	Madison, USA	2002–2013	Pre vs. post, single center	Multidisciplinary management conference	655
Serper et al^28^	VA Health System, USA	2008–2010	Retrospective, multicenter	Multidisciplinary management conference	3988
Diaz et al^29^ ^a^	Miami, USA	2012–2014	Retrospective, single center	Multidisciplinary management conference	227
Duininck et al^30^	Atlanta, USA	2009–2016	Pre vs. post, single center	Multidisciplinary management conference	204
Sinn et al^31^	Seoul, South Korea	2005–2013	Retrospective, single center	Multidisciplinary management conference	6619
Ehab et al^32^ ^a^	Tampa, USA	2014–2018	Pre vs. post, single center	Multidisciplinary management conference	259 (40% HCC)

^a^ Conference abstract.

### Quality Assessment

We used a modified National Institutes of Health Study Quality Assessment Tool to assess for risk of bias (Table 2). All studies clearly described the study objectives and patient eligibility criteria. Most studies had low risk of bias for exposure measurement; however, one study stratified patients based on count of disciplines visited as a surrogate of MDC. Most studies (n = 6) measured objective and guideline-concordant outcomes and were considered as low risk of bias. Many studies (n = 5) did not provide measures of variance, such as 95% CIs, when reporting differences in clinical outcomes between groups. A common limitation of studies (n = 6) was failure to report the length of follow-up time for outcome measurement. Many studies reporting associations between MDC and clinical outcomes did not adjust for potential confounders or were at high risk of residual confounding. For example, only 5 of 11 studies reporting survival estimates adjusted for both demographics and clinical characteristics.

**TABLE 2:** Assessment for risk of bias across studies

References	Consistent eligibility	Sample size	Time frame	Exposure measurement	Outcome measurement	Loss to follow-up	Confounders^a^
Chang et al^21^	Low	High	Low	Low	Medium	NR	High
Yopp et al^22^	Low	Low	NR	Low	Low	Low	Medium
Kani et al^23^	Low	High	NR	Low	Medium	NA	High
Chirikov et al^24^	Low	Low	Low	High	Low	NA	Low
Davison^25^	Low	High	NR	Low	Medium	NA	High
Vora^26^	Low	Low	NR	Low	Medium	NA	High
Agarwal et al^27^	Low	Low	Low	Low	Low	NA	Low
Serper et al^28^	Low	Low	Low	Low	Low	NA	Low
Diaz et al^29^	Low	Low	Low	Low	Medium	NA	High
Duininck et al^30^	Low	Low	NR	Low	Low	NA	Low
Sinn et al^31^	Low	Low	Low	Low	Low	NA	Low
Ehab et al^32^	Low	Low	NR	Low	High	NA	High

^a^ For studies reporting multiple outcomes, we assessed adjustment for confounders in survival analysis.

Abbreviations: NA, not applicable; NR, not reported.

### Proportion of Patients With Early-stage HCC

**FIGURE 1:**
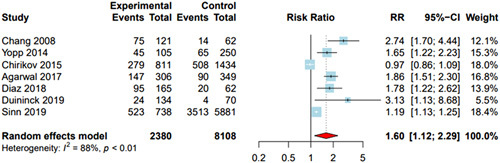
Association between multidisciplinary care and early-stage HCC. Patients under multidisciplinary care were significantly more likely to have early-stage HCC at presentation, with a pooled risk ratio (RR) of 1.60 (95% CI: 1.12–2.29). DerSimonian and Laird method was used for a random effects model.

Seven studies [n = 10,488 patients, of whom 2380 (22.7%) received MDC] reported data on tumor stage stratified by MDC status. Most studies (n = 4) used BCLC stage 0/A to define early-stage HCC, whereas 2 used the TNM staging system, and 1 used the Milan criteria (Table 3). Early-stage HCC was significantly associated with MDC, with a pooled RR of 1.60 (95% CI: 1.12–2.29) (Figure 1); however, there was significant heterogeneity (*I* ^2^ = 88%, *p* < 0.01). After excluding outlier studies (Chirikov and colleagues), sensitivity analysis revealed a similarly high heterogeneity (*I* ^2^ = 85%, *p* < 0.01) and the pooled estimate of association between early-stage presentation and MDC remained statistically significant (RR = 1.60, 95% CI: 1.15–2.23). The pooled proportions of early-stage HCC among patients who received and did not receive MDC were 47.2% (31.1%–63.8%) and 27.8% (15.1%–45.3%), respectively.

**TABLE 3:** Clinical outcomes, stratified by MDC status

References	Definition early-stage HCC	Early-stage HCC diagnosis	Curative therapy receipt^a^	Time-to-treatment initiation	Factors adjusted in survival analysis	Overall survival
Chang et al^21^	TNM stage I/II	MDC: 75/121 No MDC: 14/62	MDC: 23/121 No MDC: 4/62	NR	None	OR = 7.10 (95% CI: 3.46–14.5)
Yopp et al^22^	BCLC stage A	MDC: 45/105 No MDC: 65/250	MDC: 22/105 No MDC: 24/250	Mean 2.3 vs. 5.3 (*p* = 0.002)	Tumor stage, HCC treatment	HR = 0.40 (95% CI: 0.33–0.49)
Kani et al^23^	BCLC stage A	NR	Transplant: OR = 1.11, *p* = 0.79 Resection: OR = 1.06, *p* = 0.63	NR	None	Mean 11.1 vs. 7.4 mo (*p* = 0.01)
Chirikov et al^24^	TNM stage I	MDC: 279/811 No MDC: 508/1434	NA	NR	Demographics, comorbidities, liver etiology, tumor stage, HCC treatment	HR = 0.86 (95% CI: 0.78–0.95)^a^
Davison^25^	BCLC stage A	NR	MDC: 20.9% No MDC: 18.6%	NR	None	Median 15.1 vs. 6.3 mo (p = 0.2)
Vora^26^	Milan criteria	NR	Transplant: OR = 2.51, *p* = 0.002	NR	None	No difference 1-y overall survival
Agarwal et al^27^	Milan criteria	MDC: 147/306 No MDC: 90/349	MDC: 218/306 No MDC: 152/349	NR	Demographics, liver disease severity, AFP level, tumor stage, HCC treatment	HR = 0.72 (95% CI: 0.55–0.95)
Serper et al^28^	BCLC stage A	NR	Any treatment: OR = 1.19 (95% CI: 0.98–1.46)	NR	Demographics, comorbidities, liver disease severity, tumor stage, HCC treatment, region	HR = 0.83 (95% CI: 0.77–0.90)
Diaz et al^29^	BCLC stage A	MDC: 95/165 No MDC: 20/62	NR	No difference	None	Median 23.4 vs. 8.6 mo (*p* < 0.01)
Duininck et al^30^	BCLC stage A	MDC: 24/134 No MDC: 4/70	MDC: 5/134 No MDC: 0/70	NR	Demographics, tumor stage, HCC treatment	HR = 0.62 (95% CI: 0.40–0.98)
Sinn et al^31^	BCLC stage A	MDC: 523/738 No MDC: 3513/5881	MDC: 241/738 No MDC: 2001/5881	NR	Demographics, liver etiology, liver disease severity, tumor stage, AFP and DCP levels, HCC treatment	HR = 0.47 (95% CI: 0.41–0.53)
Ehab et al^32^	Not defined	NR	NR	Median 17 vs. 24 d (*p* < 0.01)	NA	NR

^a^ Calculated from *p*-value reported in manuscript.

Abbreviations: AFP, alpha-fetoprotein; BCLC, Barcelona Clinic Liver Cancer; DCP, des-gamma carboxy-prothrombin; MDC, multidisciplinary care; NA, not applicable; NR, not reported.

Effect size and heterogeneity were similar in subgroup analyses restricted to studies that used BCLC stage 0/A or Milan criteria to define early-stage HCC (RR = 1.60, 95% CI: 1.15–2.23, *I* ^2^ = 85%). In this subgroup of studies, pooled proportions of early-stage HCC with and without MDC were 46.9% (24.7%–70.3%) and 27.0% (9.8%–55.6%), respectively. In additional subgroup analyses, we found that results were consistent across study location, with MDC being associated with early-stage HCC in the US (RR = 1.72, 95% CI: 1.13–2.60, *I* ^2^ = 90%). Finally, MDC was also associated with early-stage HCC among studies classified as low risk of bias (RR = 1.60, 95% CI: 1.15–2.23, *I* ^2^ = 85%) but not among those classified as higher risk of bias (RR = 1.59, 95% CI: 0.002–1158.9, *I* ^2^ = 94%).

### Curative treatment receipt

**FIGURE 2:**
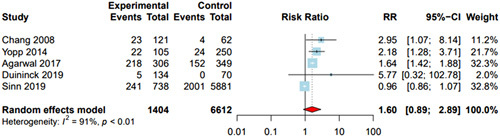
Association between multidisciplinary care and curative treatment receipt. Patients under multidisciplinary care had increased curative treatment, but the association was not statistically significant, with a pooled risk ratio (RR) of 1.60 (95% CI: 0.89–2.89). DerSimonian and Laird method was used for a random effects model.

Five studies [n = 8016 patients, of whom 1404 (17.5%) received MDC] reported data on curative treatment receipt stratified by MDC status (Table 3). MDC was positively associated with curative treatment; however, the pooled estimate was not statistically significant (pooled RR = 1.60; 95% CI: 0.89–2.89) and interpretation was limited by high heterogeneity (*I* ^2^ = 91%, *p* < 0.01) (Figure 2). Exclusion of 2 outlier studies (Agarwal and colleagues and Sinn and colleagues) led to a statistically significant association between MDC and curative treatment receipt (RR = 2.38, 95% CI: 1.34–4.23) without heterogeneity (*I* ^2^ = 0%). The pooled proportions of curative treatment receipt among patients receiving and not receiving MDC were 24.1% (5.7%–62.5%) and 10.7% (1.3%–52.5%), respectively.

Subgroup analysis by study location revealed a significant association between MDC and curative treatment receipt in the US (RR = 1.69; 95% CI: 1.37–2.09, *I* ^2^ = 0%). Finally, the association between MDC and curative treatment was not statistically significant among studies classified as low risk of bias (RR = 1.48, 95% CI: 0.73–3.02, *I* ^2^ = 93%) but was significant in the study at high risk of bias (RR = 2.95, 95% CI: 1.07–8.14).

### Time-to-treatment initiation

Three studies (n = 685 patients) reported time-to-treatment initiation, stratified by MDC status. Shorter time-to-treatment initiation was reported by Yopp and colleagues (mean 2.3 vs. 5.3 mo, *p* = 0.002) and Ehab and colleagues (median 0.6 vs. 0.8 mo, *p* < 0.01), although both were quasi-experimental studies using a pre-post design. Diaz and colleagues compared patients with and without MDC during the same study period and found no difference in time-to-treatment initiation between the 2 groups.

### OS

**FIGURE 3:**
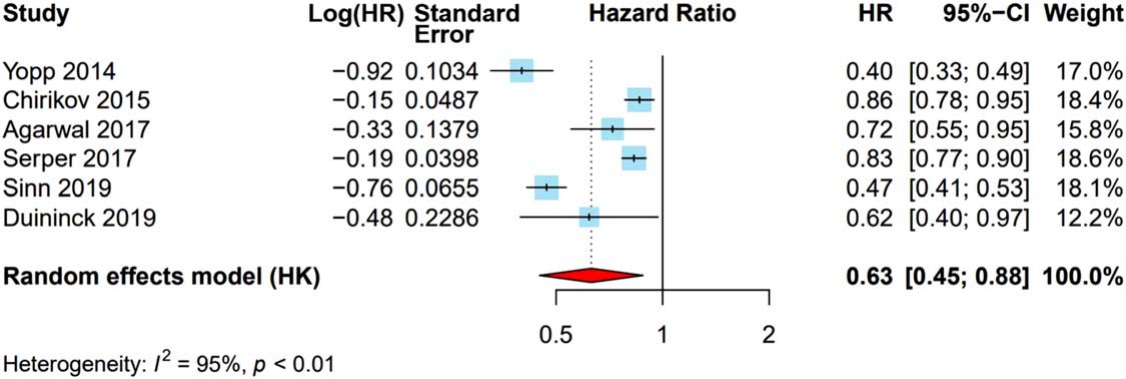
Association between multidisciplinary care and overall survival. Multidisciplinary care was significantly associated with improved survival, with a pooled HR of 0.63 (95% CI: 0.45–0.88); however, there was high heterogeneity (*I* ^2^ = 95%, *p* < 0.01). DerSimonian and Laird method was used for a random effects model.

Eleven studies (n = 15,262 patients) assessed the association between MDC and survival. There was variability in reporting of survival data, with most studies (n = 6) reporting HRs with CIs, whereas others instead reported median or mean survival (n = 3), 1-year survival (n = 1), and OR for death (n = 1) (Table 3). Among the 4 studies that reported survival outcomes for MDC using landmark analyses, Kani and colleagues reported significantly higher mean OS (11.1 vs. 7.4 mo, *p* = 0.01), Diaz et al reported significantly higher median survival (23.4 vs. 8.6 mo, *p* < 0.01), Davison et al reported higher median OS although this did not reach statistical significance (15.1 vs. 6.3, *p* = 0.20), and Vora et al found no significant difference in 1-year OS. Among the 6 studies that reported HRs for death (n = 14,066 patients, of whom 3460 were under MDC), patients managed through MDC had reduced mortality, with a pooled HR of 0.63 (95% CI: 0.45–0.88); however, there was significant heterogeneity across studies (*I* ^2^ = 95%) (Figure 3). This association remained statistically significant after exclusion of an outlier study (Yopp and colleagues) in sensitivity analysis (HR = 0.69, 95% CI: 0.50–0.95); however, heterogeneity persisted (*I* ^2^ = 94%).

MDC was associated with improved survival across subgroup analyses including studies from the US (HR = 0.67, 95% CI: 0.45–0.99, *I* ^2^ = 92%). MDC was associated with improved survival among studies classified as being at low risk of bias (HR = 0.59, 95% CI: 0.40–0.86, *I* ^2^ = 95%) and the study at high risk of bias (HR = 0.86, 95% CI: 0.78–0.95).

Four studies reported stage-stratified analyses evaluating the association between MDC and OS. Chang and colleagues found the odds of survival was greatest in patients with TNM stage II (OR = 15.5, 95% CI: 2.82–85.1) and stage IV (OR = 7.10, 95% CI: 3.46–14.5) HCC, with less benefit among those with stage I (OR = 1.44, 95% CI: 0.12–17.9) and stage III (OR = 2.19, 95% CI: 0.66–7.23) HCC. Similarly, Yopp and colleagues performed stage-stratified analyses by BCLC stage and found no significant difference in survival among patients with BCLC stage A or B HCC (*p* > 0.05 for both) but significantly improved survival with MDC in patients with BCLC stage C or D HCC (*p* < 0.001 and *p* = 0.01, respectively). Agarwal and colleagues reported that the benefit of MDC was observed in both patients with T1–T2 HCC (OR = 0.58, 95% CI: 0.37–0.92) and those with T3 or beyond (OR = 0.72, 95% CI: 0.55–0.93), whereas Kani and colleagues found the survival benefit of MDC was greatest in patients with BCLC stage A and stage B disease.

## DISCUSSION

As HCC treatment algorithms become increasingly complex, MDC models aim to promote curative treatments and optimize OS. In this systematic review and meta-analysis, we found MDC was associated with significant improvements in OS but nonsignificant increases in curative treatment receipt. Few studies reported stage-specific analyses, although results appeared consistent across tumor stages and subgroup analyses. However, currently available data are limited by potential referral bias, residual confounding, and high inter-study heterogeneity, highlighting a need for continued research.

Multidisciplinary collaborations among health care professionals were initially implemented to amalgamate the expertise of various specialists to better treat complex diseases. HCC care has become increasingly complex, given the pathophysiologic nature of the disease itself, variation in tumor biology, expanded use of surgical resection and liver transplantation, and the continuous emergence and evolution of locoregional and systemic treatment options.^30^ Studies included in our systematic review all precede the use of immune checkpoint inhibitors, which is noteworthy given the growing interest in the use of these drugs for earlier tumor stages, as well as in novel combinations with surgery and locoregional therapies.^33^ If ongoing trials demonstrate a benefit of combination therapies, MDC may be increasingly important to facilitate effective communication between providers.^9^ As silos progressively break down between treating specialties, the need for MDC for patients with cancer will likely only increase in the future. Similarly, trials and other studies to define optimal care paths, including combination treatments, would also likely recruit best in an MDC setting.

Improved OS among patients with HCC managed through MDC is likely multifactorial and could be explained by enhanced treatment discussions as well as revised imaging and biopsy interpretations for both diagnosis and appropriate staging.^31^,^34^ Although the LI-RADS criteria offer objective criteria for HCC diagnosis, radiologic interpretation has imperfect interobserver reliability.^35^ Over time, The Liver Imaging Reporting and Data System (LI-RADS) has also improved the differentiation of LR-5 (definite HCC) from LR-M (malignancy but not definite HCC), with any questionable cases being classified as the latter category and necessitating biopsy for confirmation.^36^ There has also been increasing recognition that a subset of patients have combined HCC-cholangiocarcinoma, which can affect management decisions including recommended systemic therapy and eligibility for liver transplantation.^37^

Beyond implementation of MDC, provider adherence to MDC intervention recommendations is also important to consider.^38^ A retrospective cohort study among 387 patients with HCC for whom curative treatment was recommended in a multidisciplinary meeting demonstrated that provider adherence to recommendations, which occurred in 66% of patients, was associated with reduced mortality (HR = 0.39, 95% CI: 0.27–0.54).^39^ Results from another single-center study including 137 patients with HCC similarly showed that patients who received the recommended treatment per MDC conference were more likely to undergo liver transplantation compared with those who did not receive the recommended treatment (25.6% vs. 14.4%) and had a greater 1-year survival.^38^

Although MDC cannot directly contribute to early detection, as multidisciplinary conferences only manage patients already diagnosed with HCC, we found MDC implementation is associated with a higher proportion of patients with early-stage HCC. It is possible that MDC implementation increases center-level awareness of HCC and promotes surveillance and decreases time to diagnostic resolution.^34^ This improvement could also be related to concomitant interventions from the HCC program such as provider education or focused interventions to improve HCC surveillance use.^40^–^42^ However, given most studies used a pre-post study design, observed associations may simply represent improvements in early detection over time.^43^ In addition, this association may be related to referral bias, in which patients with early-stage HCC are more likely to be evaluated in MDC settings than those with advanced-stage or terminal-stage tumors. Although patients with advanced-stage or terminal-stage HCC typically are treated with systemic therapy or best supportive care, respectively, treatment decisions in patients with liver-localized disease often have multiple treatment options that must be considered. For example, patients can be bridged or downstaged with different locoregional therapies and then be considered for liver transplantation. Future studies evaluating this association and the potential for referral bias are critical to understand the magnitude of MDC benefits in patients with HCC.

Notably, MDC implementation within health care centers can face significant limitations that may challenge its applicability, particularly in resource-limited settings.^44^ For instance, inclusion of representatives from multiple academic disciplines into weekly team meetings requires substantial use of resources, including institutional funds and additional health care personnel time per patient.^44^–^46^ However, in light of the direct benefits of MDC on HCC management, health systems with sufficient resources should consider making this investment, particularly when it is justified by a large number of cases that would benefit from it.^31^,^44^ Furthermore, to reach optimal results, a multidisciplinary team has to surmount some of the commonly encountered barriers to effective clinical decision-making.^47^ These include hierarchies and lack of trust between team members as well as organizational issues such as scheduling conflicts and lack of time to prepare for meetings.^47^ Accordingly, effective leadership of a multidisciplinary team is crucial to promote inclusiveness, resolve logistical conflicts, and subsequently ensure more favorable outcomes.^47^,^48^

We acknowledge our study has several limitations. There was high heterogeneity across pooled estimates that persisted after sensitivity and study-level subgroup analyses. Given the lack of patient characteristics data, we were unable to generate patient-level subgroup analyses that could otherwise potentially justify heterogeneity. Furthermore, larger studies generally reported a smaller effect of MDC on clinical outcomes, suggesting that high-volume centers may derive less benefit from MDC than smaller centers with less experience.^49^,^50^ In addition, we found most studies were limited by using quasi-experimental pre-post design, so some improvements in clinical outcomes may be related to independent improvements in treatment modalities in the post-MDC period. Other limitations include inherent selection and lead-time biases that could favor clinical outcomes, poor reporting of loss to follow-up, and risk of residual confounding. Finally, there were substantial variations in the functional definition of MDC across centers; thus, we were unable to compare clinical outcomes between different types of MDC, such as the incremental value of a co-located clinic beyond a multidisciplinary tumor conference.^21^,^51^ Future studies should tackle these limitations to strengthen confidence in these results.

In summary, we found a consistent association between MDC and improved clinical outcomes for patients with HCC, including OS. However, these data must be considered in light of limitations including potential referral bias and between-study heterogeneity, highlighting a need for continued research. Current evidence as well as the evolving approach to HCC treatment suggests MDC should be considered the standard of care and implemented in most health systems, provided future studies continue to generate more high-quality evidence.

## Supplementary Material

SUPPLEMENTARY MATERIAL
