# Supplementary material for: Multidisciplinary care for patients with HCC: a systematic review and meta-analysis
Source: Hepatol Commun. 2023 Apr 26;7(5):e0143. doi: 10.1097/HC9.0000000000000143 (PMC10146543; doi:10.1097/HC9.0000000000000143)
Supplement: SUPPLEMENTARY MATERIAL [file hc9-7-e0143-s001.docx]

**Supplemental Figure 1.** Results of the electronic-based literature search.

2484 citations reviewed

189 abstracts reviewed

53 full texts reviewed

7 articles included

+ 5 relevant conference abstracts

2295 citations not regarding impact of multidisciplinary care (MDC) on HCC clinical outcomes

53 studies analyzed multidisciplinary treatments, not teams

44 studies did not have original data

24 studies did not measure patient outcomes

6 studies were case reports

5 studies did not have a control group

3 studies did not measure outcomes based on MDC

1 study was not regarding HCC

11 studies did not have original data

3 studies did not measure patient outcomes

3 studies did not have a control group

3 studies did not measure outcomes based on MDC

1 study analyzed multidisciplinary treatments, not teams

1 study was a case report

1 study had dual intervention: MDC and surveillance

Full text articles to 23 abstracts could not be found
